# Educating the Educators

**Published:** 1911-02-15

**Authors:** E. A. Thomas

**Affiliations:** Red Cloud, Nebraska


### EDUCATING THE EDUCATORS.

E. A. THOMAS, D.D.S., RED CLOUD, NEBRASKA.

So much has been written upon the subject of Dental education in the last year that it would be useless for me to attempt offering anything new, so I will only give you an outline of some of the phases of this great subject as I see them. Not so much with the expectation of adding to the knowledge we already have, but to get even with the program committee who, instead of asking me the usual question, "What will you give?" wrote, "What is the title of your paper?" thus the inspiration for my title "Educating the Educators."

1. The first dental educator that I feel needs a little polishing is the Dental College in its education of the dentist. A real dental education is much more complex than is dreamed of by college matriculants who imagine that graduation brings cessation from study instead of "commencement day" and that is where they stop. The college should then give a little more attention to a wiser choice of recruits for this most sacred calling in human life, ever being cognizant of the fact that through its portals must pass the few men who make or mar the fair name of dentistry. Ever impressing the fact that those older in the profession already busy, and those gone on before, made it possible for them to reach commencement day, and that they have some claim for consideration from teachers of young dentists, and that the profession is not merely the ability to properly perform dental operations, but to become an educator and moulder of public opinion as to his profession and that his school days are not a limited few, but three hundred and sixty-five days a year, and his patients are his pupils.

2. Our next educator is the dentist. The post-graduate. The worst enemy to dental education is the dentist. The best, surest, and safest way to eliminate the quack is to educate the public so that their knowledge of the teeth and oral cavity and the service to be rendered is such that they will know when it is, or is not, rendered properly and not have to wait for a durability test to judge ability. The two factors in educating the post-graduate is through the dental society and the dental journals. I believe that the time is at hand when the societies will become more firmly united and grounded in making them stepping stones in this great post-graduate course. First, the Local or County Society should be chartered by the District Society, the District Society by the State Society, and the State Society by a National District Society, which in turn is chartered by the National Society. And a dentist's advance in these societies would be by certain qualifications which should be strictly adhered to. Make the societies stand for something in the eyes of the public, whose membership is respected and looked to with honor by his patrons. Then each National District Society to publish its journal, and the National Society its journal, and not put it on subscription, but see to it that only those qualified receive one. Thus bringing about an educational movement that would force the indolent, stay-at-home to come out of his shell. We could then demand proper legislation.

3. The physician must be brought to know and respect our particular specialty, and lend his aid to this great movement. That we are not mere mechanics or tooth pluggers, but scientific, and professional. A closer affiliations hould be brought about by getting together clubs, sending delegates from our societies to theirs, and asking them to do the same. These delegates to report to their respective societies, whether dental or medical, all proceedings of interest, etc. We could, by working in common, assist them in their public educational movement, as well as them ours.

The Newspaper. The societies are doing much to better the newspaper, and I think that every society should have an editorial committee to pass on all matter that would go to the press. To urge strictly ethical and decent write-ups, and to see that their wishes were granted. In order to find out the attitude of the press in regard to our educational matters I gave some editors a number of clippings for the press as given in the *Dental Summary* and their findings are summed up in this one letter to me.

"The Chief Publishing Co."

"RED CLOUD, NEB., February 11, 1910.

"DEAR DOCTOR:—Replying to your query regarding the publication of articles written relative to the care of the teeth, will say that upon careful examination of those articles I find that they are so composed as to admit of two distinct decisions, and yet the line of demarkation is very elusive. Either they are of vital importance to the public and therefore deserve wide circulation free of charge, or else they are clever advertisements, pure and simple. If the first premise be true it would naturally be presumed that editors would gladly spread the information to the masses, for, be it remembered, editors do more of this than any other class of people. But when one asks the question to who's good? in dollars and cents the dentist looms up to the exclusion of all others. The people cannot fill their teeth. As soon as they know that they should give their mouths careful attention they immediately think of the mouth doctor, and he receives the benefit of the article supposedly written for his general welfare. But so much for that.

"The other position assumes that they are advertisements. As such they are legitimate weapons in the hands of skilled dentists for drumming up trade. I know that I am now touching a tender spot, as your profession seeks to avoid publicity on the grounds that it smacks of quackery. Right here permit me to give you a jolt. Dentists, under the guise of safeguarding their high calling, place themselves in the attitude of 'holier than thou' and stand aloof for fear of besmirching their garments. They forget that advertising is legitimate, honorable, and, I might add, compulsory. The public has a right to know the man to whom it entrusts its sacred oral cavity. Many a man has permitted an incompetent dentist to ruin his mouth simply because he had no means of knowing the qualifications of the practitioner. In days gone by conservative banking institutions considered it the greatest error to advertise but now all that is changed. People want to know the bank, its officials, its method of doing business, and the like, and they have a right to the information. How much more important that they know the qualifications and fitness of the dentist. Dollars can be re-made, a ruined mouth never.

"No, sir, the crime of your profession is that you do not advertise. Advertisements properly written would place dentists upon a higher plane and would convince the public that there was nothing to hide, and they would soon learn to taboo the man who refused to use printer's ink. Advertisements tell the truth, and the truth will not harm the honorable. I know that some dentists have all the work they can do and more, and hence will say that they do not need to advertise, but that does not keep them from forcing the quack to keep still. Many good dentists do not have enough to do. If you are so desirous that the editor educate the people you ought to see to it that the people are protected from frauds. In conclusion I am convinced that your articles are advertisements and as such must be paid for if they appear in the columns of the Chief.

"With greatest personal respect, I am,

"Sincerely yours,

"C. B. HALE, Editor."

We must look at these matters from their point of view, and learn a lesson from them, as well as they from us. If we were organized into societies as I have stated with proper qualifications for membership, and these qualifications in the hands of a standing ethic or qualification committee and society membership allowed to insert and use on their professional cards the fact that they are members of different socieites and thereby, as the editor says, show the public who is the most up-to-date and progressive, and draw for them some demarkation between the really good and the quack.

5. The public school educator. In this educator, in my opinion, lays our greatest field and surest results, which come slowly but surely. Some have said to me that the only way to bring about public education is to legislate. We must first educate before we can hope to legislate. We must lay aside petty personal ambitions and prejudicial strife, and forget the idea that one dentist with a political bug in his hat can roll through the legislature any kind of a bill he may propose. We must educate, and the first place we must strike is at the heads of our educational institutions, we will say the State Superintendents of Public Instruction. By simple, plain, and logical facts bring them to a full realization of their duty in these matters and they respond readily. Through their co-operation send out through their office, with their endorsement to all the county superintendents and superintendents of high schools and they in turn to all the teachers and subordinates under them and they in turn to their pupils, and the pupils to their parents, and in time to their children who will become the dental students, dentists, physicians, journalists, editors, and school teachers. An evolution slow but sure. I believe much in the circular letter endorsed by proper authorities, being placed in the hands of educators with follow up letters or lessons ever so often. I have been impressed with a successful incubator manufacturer who has a series of lessons on chicken raising. Once a month he sends to prospective customers lessons number 1, number 2, number 3, etc., and they are in very plain, simple language so a child can read them, attractive and catchy. I believe we can get a good lesson from him. Say this Society prepares six or seven little talks on oral hygiene, have them printed and numbered, and address them to teachers every month during the school year with the request that they be read to their scholars, much good could be accomplished. If we had a book of a few short chapters covering the subject and get it into the state reading circle requirements with one, two or three questions from it in the teachers's examinations a greater good could be accomplished. Every local society should delegate some good talker or enthusiast as society lecturer and own its own charts or slides and by each adding a little, say one, two, or more dollars, this member could visit the principal schools in the society district and give talks or lectures, say one in the school, and one to all who would come in the evening. This to be arranged by the members in the town as they saw fit. This office of lecturer to be an honorary one and only his legitimate expenses to be paid by the society. There are only a few of the educators to be educated for we must ever bear in mind that in every walk of life in every act, no matter who the actor may be, the highest or the lowest, there is an educator who in turn must be educated.—*Western Dental Journal.*

